# *Broussonetia papyrifera* ameliorates imiquimod-induced psoriasis-like skin inflammation in mice by modulating the TLR4/NF-κB and PI3K/AKT signaling pathways

**DOI:** 10.1371/journal.pone.0322710

**Published:** 2025-05-07

**Authors:** Xiaoqiang Huang, Li Wang, Xiaoying Ma, Shunhe Liu, Hongchang Zhao, Pengbo Zhang, Liyan Li, Wanli Zhao, An Jia

**Affiliations:** 1 School of Medicine, Huanghe Science & Technology University, Zhengzhou, China; 2 School of Pharmacy, Henan University, Kaifeng, China; 3 Jiangsu Key Laboratory for the Research and Utilization of Plant Resources, Institute of Botany, Jiangsu Province and Chinese Academy of Sciences (Nanjing Botanical Garden, Mem. Sun Yat-Sen), Nanjing, China; Xiamen University - Malaysia Campus: Xiamen University - Malaysia, MALAYSIA

## Abstract

Psoriasis is a chronic, immune-mediated inflammatory skin disease, and the inflammatory response plays an important role in its development and progression. Psoriasis can appear at any age and occurs around the world. The pathogenesis of psoriasis has not been fully elucidated, and there is currently no effective treatment method in clinical practice. *Broussonetia papyrifera* is a traditional Chinese medicine that exhibited a significant therapeutic effect on psoriasis in our previous study due to its remarkable anti-inflammatory and anti-oxidant properties. However, its mechanism of action in treating psoriasis is still unclear. The purpose of this study is to evaluate the anti-psoriasis effect of the *B. papyrifera* leaves extract (PLE) in vivo and to explore its potential effects. PLE effectively alleviated imiquimod (IMQ)-induced psoriasis-like lesions, reduced psoriasis lesion area and severity index, decreased epidermal hyperplasia, ameliorated the oxidative stress-induced changes in the levels of superoxide dismutase (SOD) and malondialdehyde (MDA), and reduced the levels of the inflammatory cytokines TNF-α and IL-17A. PLE can also reduce the protein expression levels of TLR4, MyD88, p-NF-κBp65, p-IκBα, p-PI3K and p-AKT induced by IMQ model. Our findings suggest that PLE is effective in improving psoriasis-like symptoms, which might be ascribed to the inhibition of the TLR4/NF-κB and PI3K/AKT inflammation pathway. Our study demonstrates the potential mechanism of a natural source of PLE for the treatment of psoriasis. However, it is important to note that these findings lack clinical validation, and further studies are required to validate these results in clinical settings. Additionally, PLE shows potential in being a cost-effective alternative compared to existing biologics, which could have broader implications for psoriasis treatment in the future.

## Introduction

Psoriasis is an immune-mediated inflammatory skin disease characterized by common, chronic, and recrudescent [1]. Psoriasis has seriously affected the quality of life of patients and has been described by the WHO as a “chronic, non-communicable, painful, disfiguring, and disabling ailment without a definitive remedy” [2–4]. Psoriasis has been reported to affect a world population of approximately 125 million [5]. The exact pathogenesis of psoriasis is not yet clear, but it is generally believed that inflammatory response and abnormal activation of immune cell function play a key role in the occurrence and development of psoriasis. Excessive inflammatory factors activate multiple intracellular signaling pathways, stimulate transcription factors, and lead to a sharp increase in cytokine levels released by immune cells, exacerbating epidermal symptoms and ultimately worsening psoriasis. Inflammation in psoriasis is not only manifested in the skin, as skin immune cells and inflammatory cytokines circulate throughout the system, it is manifested as inflammation in other parts of the body. While at the skin level, it manifests itself as epithelial hyperplasia of the skin, hyperkeratosis, penetration of the dermis into various inflammatory cells [6,7]. Psoriasis is characterized by abnormal keratinization, epidermal hyperplasia, and inflammation [8]. Its pathogenesis is complex and existing research have identified IL-17 and IL-23 as key factors in the pathogenesis of psoriasis [9,10]. Several environmental factors, such as trauma, infections, and medications, are thought to influence the development of psoriasis [11].

Traditional drugs have poor therapeutic effects, cannot effectively solve the problem of recurrence, and have certain side effects. In recent years, although biologics targeting inflammatory factors have certain therapeutic effects, their side effects cannot be ignored. Long term use increases the risk of adverse reactions such as skin cancer and malignant tumors [12,13], and the price is expensive, which makes patients prone to resistance and poor compliance. Hence, it was urgent to find an agent with obvious therapeutic effect and low side effects in the treatment of psoriasis.

*Broussonetia papyrifera* (L.) L’Hér. ex Vent. is a perennial broad-leaved of *Broussonetia* family in *Moraceae*. *B. papyrifera*, also known as “Chu tree” in Chinese, is a perennial broad-leaved deciduous tree widely distributed throughout Asia. It is rich in vitamins, proteins, flavonoids, alkaloids, polysaccharides and other ingredients. According to the Compendium of Materia Medica, the leaves have the function of ringworm and sore. In ancient Chinese medicine, “ringworm” mostly refers to skin diseases caused by fungal infections, such as ringworm of the hand, athlete’s foot, athlete’s body, etc., while “sore” may include skin ulcers or inflammation caused by bacterial infections or other reasons. In addition, in Chinese folk, it is also useful to apply water decoction of leaves or conformation SAP to relieve skin itching and psoriasis. Earlier, the research group has also confirmed that the extract of the leaves has a alleviating effect on psoriasis [14]. The extract of *B. papyrifera* leaves were found to possess anti-oxidant, anti-inflammatory, anti-bacterial, anti-tumor and hepatotoxic properties [15–19]. Compounds extracted from B. *papyrifera* have demonstrated inhibition of NF-κB signaling in human monocytic leukemia cells (THP-1), and efficacy in suppressing the production of pro-inflammatory cytokines, including TNF-α, IL-1β, and IL-6 [20,21]. However, the mechanism of psoriasis treatment by *B. papyrifera* leaf has not been reported so far. In order to further understand the pharmacological effects of *B. papyrifera* leaf extract (PLE) and explore the potential mechanism of its therapeutic effect on psoriasis, we have carried out experiments in vivo tests. Based on the various pharmacological activities of *B. papyrifera*, this study investigated the role and potential molecular mechanisms of PLE in the treatment of psoriasis by establishing an IMQ-induced BALB/c psoriasis-like mouse model.

## Materials and methods

### Materials and reagents

Prof. Li Wang, Director of the Department of Pharmacy at Huanghe Science & Technology University, identified the specimens (voucher No. BP-2023–0812) as leaves of *Broussonetia papyrifera* (Moraceae). Standard products such as Neochlorogenic acid, Chlorogenic acid, Cryptochlorogenic acid, Orientin, Vitexin, Scutellarin, Apigenin-7-*O*-glucuronide, etc. were purchased from Shanghai Yuanye Biotechnology Co., Ltd. (purity ≥ 98%, Shanghai, China); chromatography-grade phosphoric acid (Tianjin Comio Chemical Reagent Co., Ltd.); chromatography-grade acetonitrile (Merck KGaA KGaA); chromatography-grade methanol (Merck KGaA, Germany). SOD assay kit and MDA test kit were obtained from Nanjing Jiancheng Bioengineering Institute (Nanjing, China); high-efficiency RIPA lysis buffer (tissue/cell), 5 × protein loading buffer, Bovine serum albumin was obtained from Beijing Solarbio Science & Technology Co., Ltd (Beijing, China); PBS buffer was obtained from Shanghai Darthill Biotechnology Co., Ltd (Shanghai, China); 10% SDS-PAGE gel ultra-fast preparation reagent box, protein phosphatase inhibitor mixture, Western transfer solution, SDS-PAGE electrophoresis solution, BCA protein concentration determination kit, TBST, and skimmed milk powder were purchased from Biyuntian Biotechnology (Shanghai, China); MyD88, and TLR4, AKT, phospho-AKT, PI3K, phospho-PI3K, NF-κB p65, phospho-NF-κB p65, β-Actin, IκBα, phospho-IκBα, were obtained from Chengdu Zhengneng Biotechnology Co., Ltd. (Chengdu, China); protein markers were obtained from Thermo Scientific; ultra-sensitive ECL luminescent solution was obtained from Dalian Meilun Biotechnology Co., Ltd. (Dalian, China); Imiquimod (IMQ) cream was provided by Mingxin Pharmaceutical (0.5%; Mingxin Pharmaceutical, SiChuan, China); TNF-ɑ and IL-17A ELISA kits were obtained from Lianke Biotechnology Co., Ltd. (Hangzhou, China); Depilatory Cream were obtained from Veet.

Other reagents are of analytical grade.

### Identification of the components of *B. papyrifera*

The collected fresh *B. papyrifera* leaves were dried in an oven, crushed and sieved through a 20-mesh sieve to obtain *B. papyrifera* leaf powder. The powdered *B. papyrifera* leaf was extracted after soaking in 90% ethanol at a ratio of 1:5 (kg/L) for 24 hours, and then 90% ethanol was added to extract at a ratio of 1:1 (kg/L), the extraction was repeated 6 times, and then the ethanol extract of *B. papyrifera* leaves was combined. The ethanol extract of the leaves of *B. papyrifera* were extracted with petroleum ether, and the composition obtained after removing the extracted components of petroleum ether was the extracted part of the leaves of *B. papyrifera*, hereinafter referred to as PLE (*B. papyrifera* Leaf Extract), which was used in this experiment.

The PLE components were analyzed using LC/MS. Chromatographic conditions: The chromatographic column used was a Hypersil GOLD aQ column (100 × 2.1 mm, 1.9 μm, Thermo Fisher Scientific, USA). The mobile phases were 0.1% formic acid in water (solution A) and 100% acetonitrile containing 0.1% formic acid (solution B), and the gradient elution was performed at a flow rate of 0.3 mL/min, the column temperature was 40 °C, and the injection volume was 5 μL. Mass spectrometry conditions: a Q Exactive mass spectrometer (Thermo Fisher Scientific, USA) was utilized for primary and secondary mass spectrometry data acquisition. The mass spectrometry scanning mass-to-core ratio ranged from 150 to 1500, and according to the parent ion intensity, Top3 was selected for fragmentation to collect secondary information. Ion source (ESI) parameter settings: Sheath gas flow rate gas flow is 40, Auxiliary gas flow rate is 10, Spray voltage for positive ion mode was 3.80, negative ion mode was 3.20. Capillary temperature was 320 °C, and Auxiliary gas heater temperature was 350 °C. The LC-MS/MS data were processed using Compound Discoverer 3.1 (Thermo Fisher Scientific, USA) software, mainly for peak extraction, peak alignment and compound identification.

### *B. papyrifera* leaf extract content determination

Meanwhile, we used HPLC method to determine the contents of neochlorogenic acid, chlorogenic acid, cryptochlorogenic acid, orientin, vitexin, scutellarin, and apigenin-7-*O*-glucuronide in PLE. Chromatographic conditions: Chromatographic column: Agilent ZORBAX SB-C18 column (4.6 × 250 mm, 5 μm), mobile phase: acetonitrile-0.1% phosphoric acid water, gradient elution (0 ~ 15 min, 10% acetonitrile; 15 ~ 20 min, 10 ~ 20% acetonitrile; 20 ~ 25 min, 20% acetonitrile; 25 ~ 40 min, 20 ~ 30% acetonitrile; 40 ~ 45 min, 30 ~ 45% acetonitrile; 45 ~ 50 min, 45 ~ 60% acetonitrile;, detection wavelength: 339 nm, The flow rate was 1 mL/min, the injection volume was 5 μL, and the column temperature was 25 °C. Preparation of sample solution: Take 0.5g of leaf powder (passed through No. 2 sieve), weigh it accurately, place it in a stoppered Erlenmeyer flask, add 25 mL of 60% ethanol accurately, weigh, sonicate for 30 minutes, cool, and replenish with 60% ethanol. Weigh, shake well, filter through 0.45 μm filter membrane, and take the remaining filtrate for HPLC measurement.

### Animal experiments

Experimental animals were provided by the Animal Experiment Center of the School of Medicine, Huanghe Science & Technology University. Approval for all experimental procedures was obtained from the Animal Ethics Committee of Huanghe Science & Technology University (approval no. 2023-011). The male BALB/c mice (20 ± 2 g) of seven-week-old were feed in a normal environment with food and water. On the day before the experiment initiation, perform dorsal depilation by first conducting a coarse shave using an electric shaver, followed by gentle and even application of Veet depilatory cream to the shaved area on the mouse’s back to remove residual hair. After substantial hair removal, use a moistened clean cotton ball to wipe away residual depilatory cream from the mouse’s back, creating an exposed skin area approximately 2 cm × 3 cm in size [22].

The mice were divided into the following five groups, with 9 mice in each group (n = 9): control group (Ctrl, treated with petroleum jelly); model group (IMQ, treated with imiquimod); PLE low, medium, high dose groups. The mice in the control group were smeared with Vaseline on the depilated area on their backs once a day, and the mice in the model group were smeared with 62.5 mg of 5% imiquimod (Sichuan Mingxin Pharmaceutical Co., Ltd.) on the depilated area at the same time every day to induce the psoriasis-like symptoms for eight consecutive days [23]. Following 6 hours of IMQ treatment, mice were treated with different doses of PLE mixture. Our prior research has demonstrated that the extract derived from PLE therapeutic efficacy in the treatment of psoriasis [14]. So, the corresponding administration groups of mice were categorized into low-dose group (PLE L, IMQ + 50 mg/kg PLE L), medium-dose group (PLE M, IMQ + 100 mg/kg PLE M), and high-dose group (PLE H, IMQ + 200 mg/kg PLE H).

### PASI score

From the first day of the animal experiment, according to the PASI scoring standard [24], three scores were scored on the exposed back area of the mice every day: erythema, scale and infiltration, asymptomatic 0 points, mild 1 point, moderate 2 points, severe 3 points, Extremely serious is 4 points, and the sum of the three items is the total PASI score.

### Histological examination

After the PASI scores were recorded on the eighth day, the mice were sacrificed by cervical dislocation, and skin samples of 0.5 cm × 0.5 cm were taken and immediately fixed in tissue fixative, followed by H&E staining [25].

### Determination of oxidative stress factors

Commercial kits for superoxide dismutase (SOD, A001-3) and malondialdehyde (MDA, A003-1) were procured from Nanjing Jiancheng Institute of Biology (Nanjing, China) to evaluate oxidative stress levels in psoriatic tissue or serum of mice. Skin tissue samples were extracted for protein, and the levels of the aforementioned oxidative stress factors were assessed using the kit according to the manufacturer’s instructions.

### Enzyme-linked immunosorbent assay (ELISA)

The skin tissue of psoriatic-like mice was homogenized and centrifuged, and a standard curve was established according to the kit instructions, and the OD value was detected with a microplate reader to calculate the level of related inflammatory factors (IL-17A, TNF-α). TNF-ɑ and IL-17A ELISA kits were obtained from Lianke Biotechnology Co., Ltd. (Hangzhou, China).

### Western blot analysis

Skin tissue protein extraction [26]: Weigh a certain amount of skin tissue, cut it into fragments, transfer all the chopped sample tissue to a pre-cooled tissue homogenizer, and add protein lysis solution (high-efficiency RIPA lysis solution: Protease inhibitor: Phosphatase inhibitor = 100:1:1) that is 10 times the tissue weight, grind thoroughly on ice, then transfer to a pre-cooled centrifuge tube, centrifuge at 1200 rpm for 10 minutes at 4 °C, transfer the supernatant to a pre-cooled (be careful not to absorb fat). Protein quantification was performed using the BCA method.

Before use, all samples were adjusted to the same concentration using 5 × loading buffer, boiled in water until the protein was fully denatured, and centrifuged briefly before use. Protein samples were separated on a 10% SDS-PAGE gel and transferred to a 0.45 μm polyvinylidene fluoride (PVDF) membrane, and then the PVDF membrane was blocked with 5% skim milk or 5% bovine serum albumin for 2 h. The blocked PVDF membrane was incubated with primary antibodies (PI3K, p-PI3K, AKT, p-AKT, IκBα, p-IκBα, NF-κB P65, p-NF-κB P65, TLR4, MyD88, COX-2, β-Actin, Zhengneng Biotechnology Co., Ltd., China) incubated overnight at 4 °C. The PVDF membrane was washed 3 times with 1 × TBST on a shaker, 10 min each time. The membrane was then incubated with HRP-labeled secondary antibodies for 1 h at room temperature, and the immunobands were visualized using chemiluminescent reagents in the Chemi-Doc XRS system, and the gray value of the protein bands was analyzed by Image J software.

### Statistical analysis

GraphPad Prism 8.0 software was used for data analysis. The data were expressed as the mean ± standard deviation of each group. One-way ANOVA was used for statistical analysis between different groups. The results of *P < *0.05 were considered to be significantly different and statistically significant.

## Results

### LC-MS results of *B. papyrifera* leaf extract

**Fig 1:**
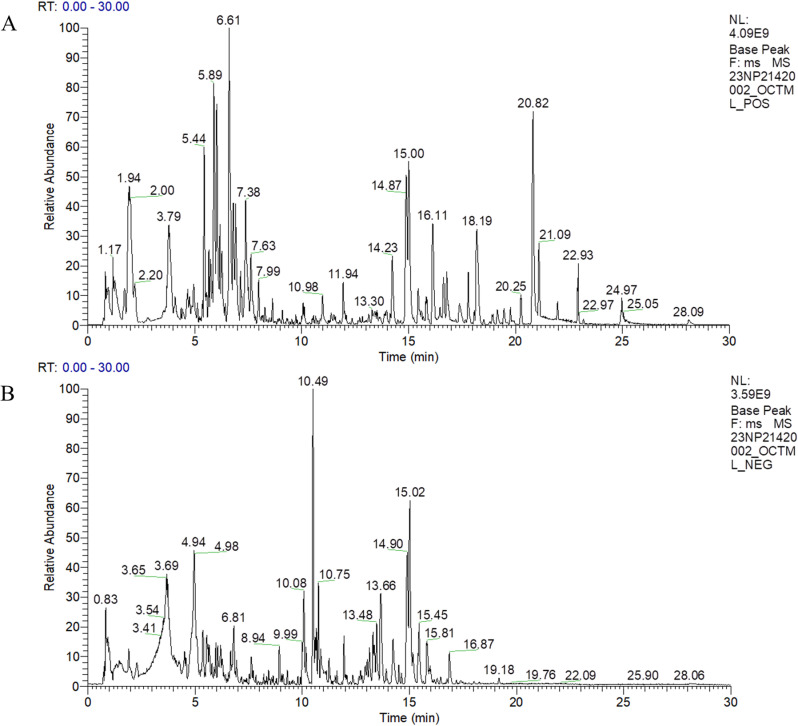
(A) and (B) represent the total ion chromatogram in positive and negative model of qualitative analysis, respectively.

The components of *B. papyrifera* leaf extracts were analyzed by LC-MS (Fig 1A and 1B). The raw mass spectra data acquired via LC-MS (Liquid Chromatograph-Mass Spectrometer) were imported into Compound Discoverer 3.1 (Thermo Fisher Scientific, USA) for subsequent processing and analysis, and a total of 4,587 compounds, including flavonoids, terpenoids, alkaloids and organic acids, were identified by database comparison. Some of the major components in PLE are listed in Table 1.

**Table 1:** LC/MS composition of *B. papyrifera* leaves extract.

Compound Name	Formula	Calc *m/z*	RT(min)	Type
Apigenin	C_15_H_10_O_5_	271.05991	10.175	Flavonoids
Orientin	C_21_H_20_O_11_	449.1079	6.295	
Apiin	C_26_H_28_O_14_	565.1546	7.467	
Daidzin	C_21_H_20_O_9_	417.11768	6.528	
Cynaroside	C_21_H_20_O_11_	449.10742	7.015	
Luteolin 7-glucuronide	C_21_H_18_O_12_	463.08676	7.01	
Vitexin	C_21_H_20_O_10_	433.11228	6.701	
Isovitexin	C_21_H_20_O_10_	433.11224	6.849	
Genkwanin	C_16_H_12_O_5_	285.07562	12.784	
Apigenin 7-*O*-glucuronide	C_21_H_18_O_11_	447.09186	7.675	
Naringenin	C_15_H_12_O_5_	271.06091	9.864	
Eriodictyol	C_15_H_12_O_6_	287.05585	8.787	
Vicenin ii	C_27_H_30_O_15_	593.15118	5.646	
Hydroxygenkwanin	C_16_H_12_O_6_	299.05591	11.405	
Homoorientin	C_21_H_20_O_11_	447.09271	6.208	
Vitexin rhamnoside	C_27_H_30_O_14_	577.15637	6.662	
Homoplantaginin	C_22_H_22_O_11_	461.10852	7.789	
4’-*O*-glucosylvitexin	C_27_H_30_O_15_	593.15125	6.435	
8-prenylnaringenin	C_20_H_20_O_5_	339.12375	13.557	
Vaccarin	C_32_H_38_O_19_	725.19653	5.411	
Vicenin iii	C_26_H_28_O_14_	563.14099	6.691	
Luteolin	C_15_H_10_O_6_	285.04025	9.228	
Kaempferol	C_15_H_10_O_6_	285.04028	10.251	
Mulberrin	C_25_H_26_O_6_	421.16522	16.346	
Liquiritigenin	C_15_H_12_O_4_	255.06604	10.995	
Neohesperidin	C_28_H_34_O_15_	609.18433	6.807	
5,7-dihydroxychromone	C_9_H_6_O_4_	177.01938	7.293	
Poncirin	C_28_H_34_O_14_	593.18768	7.522	
Calycosin	C_16_H_12_O_5_	283.06082	12.989	
Taxifolin	C_15_H_12_O_7_	303.05063	6.914	
Baicalin methyl ester	C_22_H_20_O_11_	459.09299	8.094	
Formononetin	C_16_H_12_O_4_	269.08096	11.037	
Diosmetin	C_16_H_12_O_6_	301.07047	10.405	
Chrysin	C_15_H_10_O_4_	255.06533	12.45	
Prunin	C_21_H_22_O_10_	435.12845	7.549	
Lysionotin	C_18_H_16_O_7_	345.09647	13.416	
Wogonin	C_16_H_12_O_5_	285.07559	13.029	
Maslinic acid	C_30_H48O_4_	455.35251	16.512	Terpenoids
Ursolic acid	C_30_H48O_3_	439.35699	19.841	
Curdione	C_15_H_24_O_2_	237.18499	13.077	
Betulin	C_30_H_50_O_2_	443.3884	22.116	
Bryodulcosigenin	C_30_H_50_O_4_	497.36316	18.214	
Alisol b 23-acetate	C_32_H_50_O_5_	515.37408	18.275	
Carnosol	C_20_H_26_O_4_	329.17551	14.551	
Bilobalide	C_15_H_18_O_8_	325.09275	4.858	
Geniposide	C_17_H_24_O_10_	387.12967	5.156	
Eleutheroside e	C_34_H_46_O_18_	787.27063	6.226	
Secoxyloganin	C_17_H_24_O_11_	403.12439	6.017	
Protodioscin	C_51_H_84_O_22_	1047.54089	15.765	
Genipin	C_11_H_14_O_5_	207.06619	8.398	
Loganin	C_17_H_26_O_10_	435.15082	4.52	
Atractyloside a	C_21_H_36_O_10_	493.22839	9.252	
Crocin ii	C_38_H_54_O_19_	859.32965	7.101	
Trigonelline	C_7_H_7_NO_2_	138.05525	0.989	Alkaloids
Stachydrine	C_7_H_13_NO_2_	144.10214	1.258	
Codeine	C_18_H_21_NO_3_	300.15878	6.849	
Morphine	C_17_H_19_NO_3_	286.14349	7.716	
4-methyl-5-thiazoleethanol	C_6_H_9_NOS	144.04816	1.807	
Gelsemine	C_20_H_22_N_2_O_2_	323.17557	8.913	
Oxymatrine	C_15_H_24_N_2_O_2_	265.19131	11.529	
Aconine	C_25_H_41_NO_9_	500.28516	6.764	
Citric acid	C_6_H_8_O_7_	191.01971	1.167	Organic acids
Maleic acid	C_4_H_4_O_4_	115.00376	1.265	
Succinic acid	C_4_H_6_O_4_	117.01941	1.261	
Adipic acid	C_6_H_10_O_4_	145.0507	3.127	
Malonic acid	C_3_H_4_O_4_	103.00372	1.148	
Suberic acid	C_8_H_14_O_4_	173.08189	6.434	
Myristyl sulfate	C_14_H_30_O_4_S	293.17893	15.952	
D-(+)-malic acid	C4H6O5	133.01436	0.863	
2-butoxyacetic acid	C_6_H_12_O_3_	131.07146	4.779	

### *B. papyrifera* leaf extract content determination of ingredients

**Fig 2:**
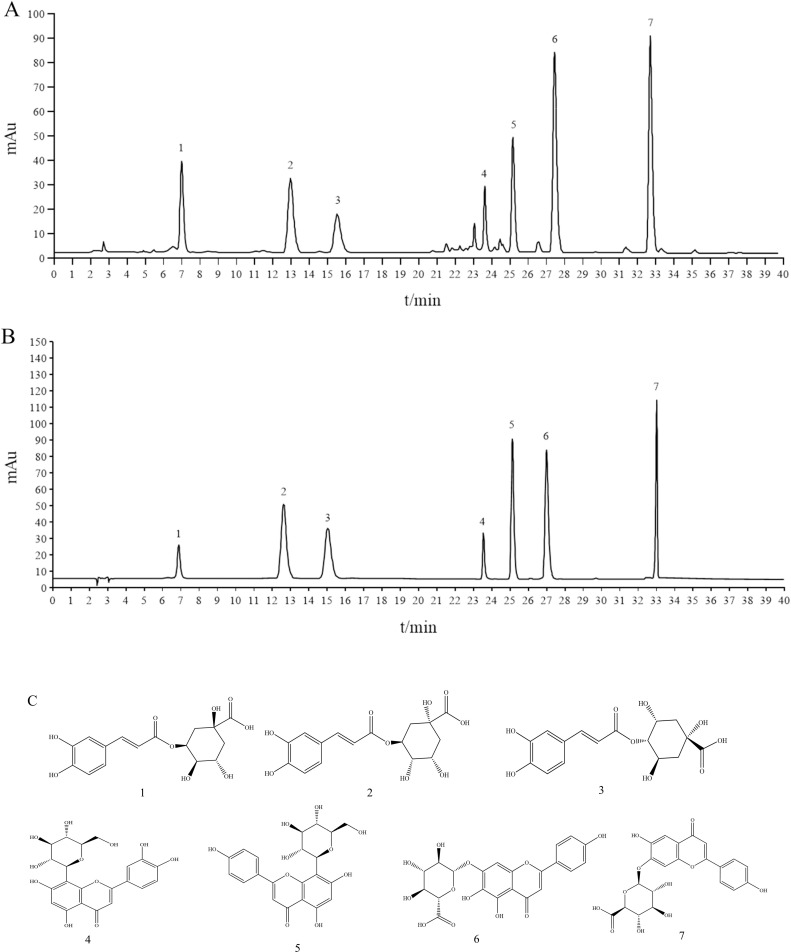
Chromatogram results of quantitative and qualitative analysis the PLE. (A) and (B) represent the chromatograms of sample and reference substance for quantitative analysis. C is the structural formula of each molecule. 1, Neochlorogenic acid; 2, Chlorogenic acid; 3, Cryptochlorogenic acid; 4, Orientin; 5, Vitexin; 6, Scutellarin; 7, Apigenin-7-*O*-glucuronide.

The contents of the *B. papyrifera* leaf were analyzed using HPLC. neochlorogenic acid, chlorogenic acid, cryptochlorogenic acid, orientin, vitexin, scutellarin and apigenin-7-*O*-glucuronide were detected and quantified. The prepared *B. papyrifera* leaf sample solution and the reference solution were tested by HPLC together. The results are shown in Fig 2A and 2B. In addition, Fig 2C is the structural formula of each molecule. Concurrently, the content of each component was computed utilizing the area normalization method. The outcomes are delineated in Table 2.

**Table 2:** Determination results of component content of *B. papyrifera leaf.*

Component	content (μg/g)
Neochlorogenic acid	5.78 ± 0.03
Chlorogenic acid	2.10 ± 0.04
Cryptochlorogenic acid	1.66 ± 0.01
Orientin	0.82 ± 0.01
Vitexin	1.42 ± 0.02
Scutellarin	2.56 ± 0.03
Apigenin-7-*O*-glucuronide	6.04 ± 0.04

### Establishment of psoriasis mouse model and drug administration

**Fig 3:**
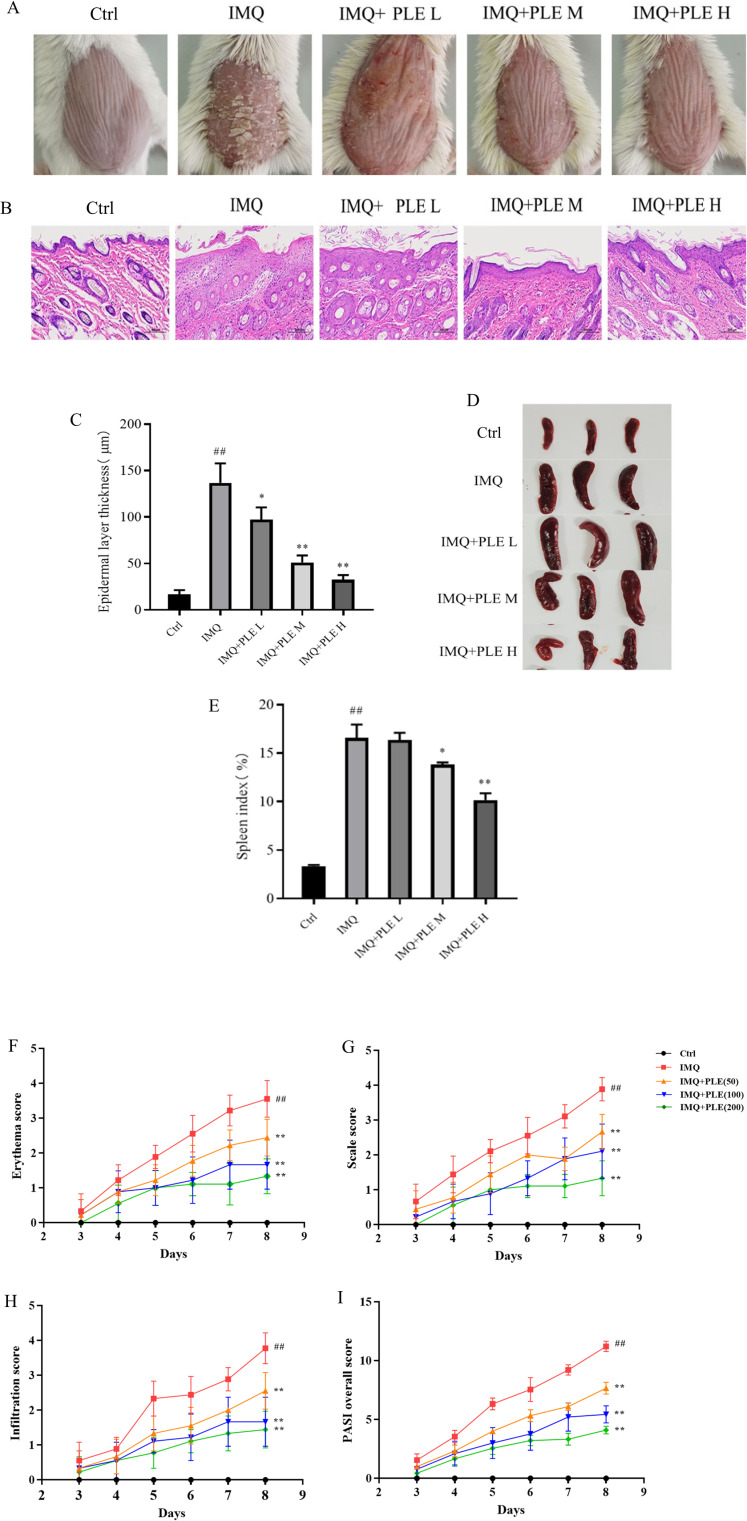
(A) Appearance image of psoriasis on the back skin of mouse. (B) Pathological examination of mouse skin tissue sections: hematoxylin-eosin staining (H&E staining). (C) Average thickness of mouse skin epidermis (μm). (D) Appearance of mouse spleen. (E) Mouse spleen gland index (%). Results are expressed as mean±SD. (F-I) Psoriasis severity assessment was performed throughout the course of the animal experiment. The degree of scale, erythema, and infiltration of the mouse back skin was scored from the third day after the scale appeared, and the score range was 0-4. Data are expressed as mean ± SD; n = 9 for each experimental group. ^*##*^*p* < 0.01 vs Control grou*p*. ^*^p < 0.05, ^**^p < 0.01 vs IMQ grou*p*.

During the mouse modeling, the exposed area on the back of the mouse was photographed and recorded. Compared with the control group, discernible impairment was observed in mice belonging to the IMQ group on the third day, mainly manifested by the appearance of fine white scales on their backs. As the modeling time increased, the worsening of skin damage severity in the mice occurred and their mental state deteriorated. The skin lesions of mice in each experimental dose group exhibited varying degrees of improvement compared to the IMQ group, mainly as a reduction in scales and a reduction in skin wrinkles. The results are shown in Fig 3A.

In order to observe the status of the mouse skin lesions more clearly and intuitively, pathological sections were made from the skin lesions on the back of the mice and H&E stained. The skin structure of the control group was intact and the cell morphology was normal. Compared with the control group, the IMQ group showed parakeratosis, the granular layer was thinned or even disappeared, the spinous layer was thickened, the boundary between the basal layer and the dermis was unclear, lymphocyte infiltration was visible in the superficial dermis and papilla, and the stratum corneum is significantly thickened and accompanied by severe inflammation indicating successful modeling of psoriasis mice. The administration group of mice exhibited a notable reduction in epidermal parakeratosis compared to the IMQ group, the spinous layer became thinner and tended to normal, the epidermal pestle shape extension phenomenon was significantly reduced or disappeared, lymphocyte infiltration in the superficial dermis was significantly reduced, and the dilation of blood vessels and disappearance of bleeding phenomena can be seen in the superficial dermis. The results above indicate that PLE can mitigate the symptoms of psoriasis-like skin lesions induced by IMQ in mice by suppressing epidermal cell proliferation and inflammatory cell infiltration (Fig 3B and 3C).

Psoriasis patients are generally accompanied by damage to the spleen. As an immune organ, the spleen is not only the place where mature T cells and B cells settle, but also the site where these cells undergo immune responses to antigen stimulation. Therefore, changes in its morphology can reflect the degree of psoriasis-like symptoms. The spleen of mice in the IMQ group exhibited significant enlargement compared to the control group, with a notably elevated spleen index. With escalating drug dosage, a corresponding reduction in spleen index was observed in the treatment group. These findings suggest a certain degree of protective effect of PLE on immune organs (Fig 3D and 3E).

Throughout the modeling period, the skin condition of mice in the control group exhibited no significant alterations, as indicated by the PASI score results, and the PASI score always remained 0. Starting from the 3rd day, symptoms such as scales and erythema appeared on the dorsal skin of mice in the remaining four groups. As the modeling time was extended, the degree of skin damage gradually worsened. The IMQ group exhibited the highest PASI score and the most severe degree of skin damage. Compared with the IMQ group, the psoriasis symptoms of the mice in the drug administration group were alleviated, and the PASI score was reduced (Fig 3F–3I).

### Effects of PLE on SOD and MDA in serum and skin lesions of psoriasis-like mice

**Fig 4:**
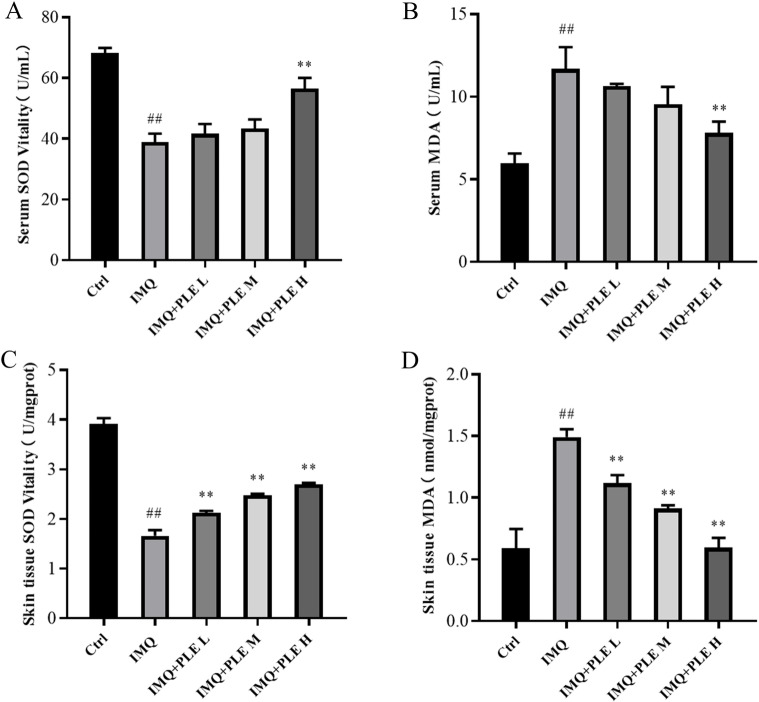
PLE inhibits IMQ-induced oxidative stress in mice, and the contents of SOD (A, C) and MDA (B, D) in mouse serum and back skin lesions were measured. Data are expressed as mean ± SD; n = 9 for each experimental group. ^*##*^p < 0.01 vs Control group. ^*^p < 0.05, ^**^p < 0.01 vs IMQ group.

The results show (Fig 4A–4D) that compared with the control group, the SOD activity in the serum and skin lesions of mice in the IMQ group was significantly reduced, The SOD activity was higher in the drug administration group compared to the IMQ group, while the MDA content in the skin lesions of mice in the IMQ group markedly exceeded that of the normal group. Post PLE treatment, there was a reduction in MDA levels. These results indicate that PLE can increase SOD activity and reduce MDA level in IMQ-induced psoriasis-like mice, and PLE can effectively regulate the oxidative/ antioxidative status of IMQ-induced mice to achieve a more favorable physiologic balance.

### Effect of PLE on the levels of inflammatory factors (TNF-α, IL-17A) in skin lesion tissue of psoriasis-like mice

**Fig 5:**
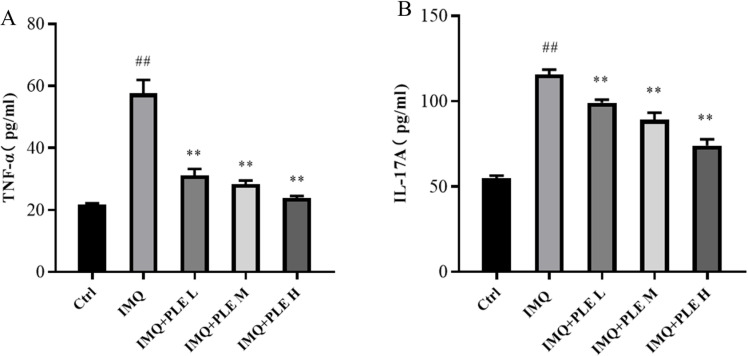
PLE inhibits IMQ-induced inflammatory in mice, and the contents of TNF-α (A) and IL-17A (B). Data are expressed as mean ± SD; n = 9 for each experimental group. ^*##*^*p* < 0.01 vs Control group. ^*^p < 0.05, ^**^p < 0.01 vs IMQ group.

Studies in immunology and genetics have pinpointed IL-17 and IL-23 as pivotal elements in the development of psoriasis. This association may be attributed to the capability of cytokines like TNF-α and IL-17A to stimulate the excessive proliferation of keratinocytes and the infiltration of immune cells. According to ELISA test findings, the expression of inflammatory cytokines TNF-α and IL-17A in the skin tissue supernatant of mice in the model group exhibited a significant increase compared to the control group, PLE significantly inhibited the release of these inflammatory factors, this result suggests that PLE can improve inflammatory damage in IMQ-induced psoriasis mice (Fig 5A and 5B).

### Effect of PLE on protein expression in skin lesions of mice with psoriasis

**Fig 6:**
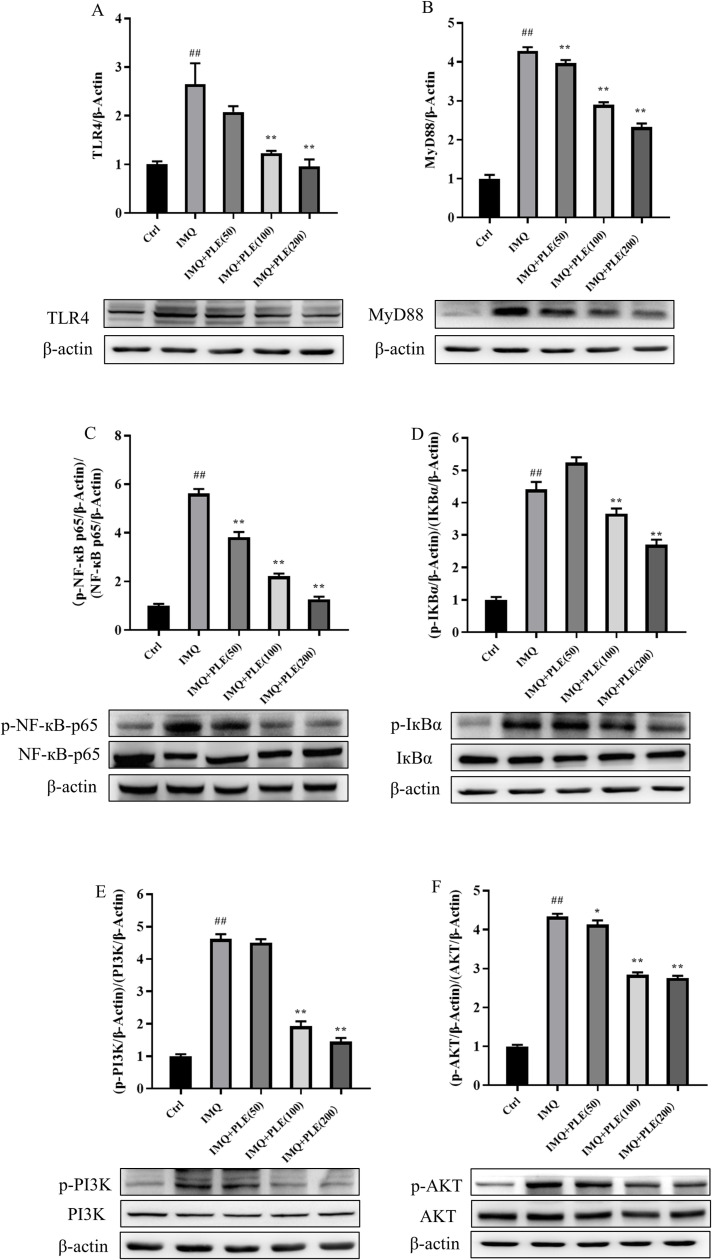
PLE prevents the development of psoriasis by inhibiting the activation of TLR4/NF- κB and PI3K/AKT signaling pathway. (A, B, C and D) Western Blot analysis to detect the protein expression levels of TLR4, MyD88, NF- κB p65, p-NF- κB p65, IκBα and p- IκBα. (E, F) Western Blot analysis to detect the protein expression levels of PI3K, p-PI3K, AKT and p-AKT. Data are expressed as mean ± SD; n = 3 for each experimental group. ^*##*^*p* < 0.01 vs Control group. ^*^p < 0.05, ^**^p < 0.01 vs IMQ group.

Based on the above results, we used Western blot to detect the expression of signaling pathway-related factors such as TLR4/NF-κB and PI3K/AKT in IMQ-induced skin tissue to further reveal the anti-psoriasis effect of PLE. Western blot results showed that IMQ promoted the expression of TLR4, MyD88, p-NF-κB p65 and p-IκBα, while PLE could reduce the protein levels of TLR4, MyD88, p-NF-κB p65 and p-IκB α in the skin of IMQ-induced psoriasis mice (Fig 6A–6D). Consistent with the above results. The findings suggest that PLE hampers psoriasis progression by hindering the activation of the TLR4/NF-κB signaling pathway. At the same time, in comparison to the control group, the protein expression levels of p-AKT and p-PI3K in the skin lesion tissues of mice in the IMQ group exhibited a significant increase, as illustrated in Fig 6E and 6F. Conversely, the protein expression of p-AKT and p-PI3K in the skin lesions of mice in the administration group showed a notable reduction compared to the IMQ group. The findings suggest that PLE treatment of psoriasis is also associated with the PI3K/ AKT signaling pathway.

## Discussion

Using the LC-MS/MS method, a total of 4587 compounds were identified, including flavonoids, terpenoids, alkaloids and organic acids. A large number of studies have confirmed that flavonoids, terpenoids, alkaloids and organic acids all have strong antioxidant and anti-inflammatory activities [26–33]. In addition, studies have reported that flavonoids and alkaloids also fight psoriasis [33–35]. The above suggests that the active component of the *B. papyrifera* leaves resistance against psoriasis may be flavonoids and alkaloids. At the same time, the content of seven main components, neochlorogenic acid, chlorogenic acid, cryptochlorogenic acid, orientin, vitexin, scutellarin and apigenin-7-*O*-glucuronide in *B. papyrifera* leaves were determined using the HPLC method. Our data show that the concentrations were determined to be 5.78 ± 0.03 μg/g, 2.10 ± 0.04 μg/g, 1.66 ± 0.01 μg/g, 0.82 ± 0.01 μg/g, 1.42 ± 0.02 μg/g, 2.56 ± 0.03 μg/g, and 6.04 ± 0.04 μg/g for neochlorogenic acid, chlorogenic acid, cryptochlorogenic acid, orientin, vitexin, scutellarin and apigenin-7-*O*-glucuronide, respectively. It has been reported that neochlorogenic acid, chlorogenic acid, cryptochlorogenic acid, orientin, vitexin, scutellarin and apigenin-7-*O*-glucuronide possess anti-inflammatory and antioxidant activities [36–42]. Furthermore, studies have demonstrated that chlorogenic acid, cryptochlorogenic acid exhibit therapeutic potential in alleviating psoriasis symptoms [43,44].

Psoriasis is a common chronic inflammatory and proliferative skin disease, characterized by abnormal proliferation and differentiation of keratinocytes and immune T cells in the epidermal layer. However, the pathogenesis of psoriasis is still not fully understood, and there is a lack of effective drugs and therapeutic strategies. IMQ activates Toll receptors (TLRs) in vivo as a Toll receptor agonist, induces localized scaly and hyperproliferative erythema resembling human psoriatic skin lesions when its cream formulation is applied topically to the skin [45–47]. Currently, researchers have successfully established an IMQ-induced psoriasis-like dermatitis animal model in mice by topically applying IMQ cream to the dorsal skin [48]. The IMQ-induced animal model not only exhibits clinical manifestations resembling psoriasis but also demonstrates characteristic histopathological features including hyperkeratosis with parakeratosis, inflammatory cell infiltration in the dermis, vascular dilation and proliferation, along with elevated expression of psoriasis-related inflammatory cytokines in lesional skin. These comprehensive pathological parallels have established this model as a high-fidelity representation of human psoriatic pathology, and it is now widely employed in preclinical studies of psoriasis.

BALB/c mice are the most extensively utilized animal models in oncology, inflammation, and autoimmune research, demonstrating heightened susceptibility to imiquimod (IMQ)-induced psoriasis-like dermatitis [49]. Compared to strains such as C57BL/6, BALB/c mice develop more pronounced cutaneous inflammatory responses under IMQ stimulation, manifesting as erythema, scaling, and epidermal hyperplasia (histopathological features mirroring human psoriasis, including hyperkeratosis and neutrophil microabscess formation). Although C57BL/6 mice are preferred for genetic manipulation studies, their weaker phenotypic response to IMQ induction often necessitates higher dosages or prolonged treatment durations [50]. These pathophysiological distinctions substantiate our selection of BALB/c mice for establishing this experimental model.

In the present study, we induced localized psoriasis-like symptoms in mice with IMQ and found that IMQ induced severe erythema, scaling, and localized infiltration. IMQ mice showed a significant reduction in psoriasis-like symptoms after application of PLE, including a decrease in all PASI scores, a decrease in the white scaly material of the skin’s surface layer, and a reduction of epidermal thickening, in conclusion, PLE had a protective effect against psoriasis-like symptoms.

The spleen is an important immune organ of the organism and is a visual indicator of nonspecific immunity of the organism. The spleen can produce lymphocytes, monocytes and plasma cells, the immune function of the organism can be assessed by the splenic index. IMQ, as a TLRs agonist, can activate the innate immune system, followed by the activation of the adaptive immune system, which subsequently leads to systemic inflammation, such as splenomegaly [51]. Consistent with the reported results, the splenic index of mice in the IMQ group was significantly elevated, and notably, the application of PLE significantly down-regulated the splenic index of psoriasis-like mice. The experimental results indicated that PLE could inhibit the recruitment of immune cells in the spleen, reduce IMQ-induced skin inflammation, and to a certain extent, it had a protective effect on immune organs.

Oxidative stress is produced by an imbalance between the levels of reactive oxygen species (ROS) and antioxidants. Dysregulation of the antioxidant system and increase in ROS is one of the major causes of psoriasis pathogenesis [52]. In addition, researches have shown that the antioxidant capacity in psoriasis patients is diminished compared to healthy individuals [53]. The stimulation of adverse external factors increases the amount of ROS production in the organism, and oxidative stress is initiated, this process results in the oxidative modification of biologically active cellular components due to the secretion of autoantigens and pro-inflammatory cytokines. It is reported that immune and inflammatory cells secrete various inflammatory cytokines during the development of psoriasis [54]. Oxidative stress also promotes inflammation through signaling pathways such as NF-κB [55]. Our data suggest that IMQ application upregulated the increase in MDA levels and downregulated the decrease in SOD levels, and that treatment with PLE resulted in a remission of these metrics, including a downregulation of MDA levels and an increase in SOD levels. In addition, we found that IMQ resulted in upregulation of TNF-α and IL-17A levels, and PLE suppressed their increase. In conclusion. Our results suggest that PLE may prevent psoriasis-like lesions by suppressing oxidative stress and inflammation.

The TLR4/NF-κB signaling pathway is an important pathway in the body’s inflammatory system and is involved in the development and regulation of a variety of diseases [56]. TLR4 is a family of innate immune recognition receptors that play an important role in initiating innate immune and inflammatory responses [57]. Cells rely on TLRs to recognize bacterial, viral, or spirochete infections and initiate the body’s immune and inflammatory responses [58]. At present, at least 11 human TLRs have been found, which are widely expressed on a variety of natural immune cells, as well as on the surfaces of keratinocytes, vascular endothelial cells, and bronchial mucosa epithelial cells. Among them, TLR4 was the earliest discovered and most extensively studied. It is the main receptor for identifying pathogenic microorganisms and their unique cell wall structures - highly conserved pathogen associated molecular patterns (PAMPs) [59]. PAMPs are essential and conserved structures for microbial survival or pathogenicity, and the most typical PAMPs are lipopolysaccharides (LPS) from Gram negative bacteria [60]. However, intestinal barrier damage and structural destruction caused by various reasons can lead to increased intestinal permeability and “Leaky Gut Syndrome”. The toxic intestinal metabolite LPS can penetrate the intestinal barrier and invade lymph vessels or blood vessels, and enter the systemic circulation through the mesenteric lymph node or portal vein, activating the immune inflammatory response of the body and distant organs (such as skin) [61].

LPS is the most important lipid component on the outer membrane of Gram-negative bacteria and is a common ligand of TLR4. Therefore, it is speculated that the increased expression of TLR4 may be due to secondary bacterial infection induced by scratches during the progression of dermatitis. TLR4 is also involved in the MyD88-dependent signaling pathway, which recruits the TIRAP/MyD88 complex and its downstream kinase IRAK1/IRAK4 complex, thereby activating NF-κB and ultimately leading to inflammation and immunological responses [62]. NF-κB is a key effector of the inflammatory response associated with various skin diseases such as psoriasis, and NF-κB has been shown to be involved in the dysregulation of psoriasis [47]. Under normal circumstances, NF-κB generally exists as a homo- or heterodimer, the most common being the p50-p65 heterodimer. In resting cells, the p50-p65 heterodimer is in an inactive state in the cytoplasm due to the formation of a trimer complex with the inhibitory protein IκB. Once stimulated by LPS or pro-inflammatory cytokines (such as TNF-α, IL-17A, etc.), the receptor proximal protein is activated and IκB is degraded, resulting in the release and translocation of p65/p50 dimers, making NF-κB dimers The polymer exposes the nuclear localization site, and the NF-κB dimer is further activated through various post-translational modifications. Free NF-κB quickly translocates to the nucleus and binds to the specific κB sequence, thereby activating NF-κB pathway, promoting the expression of pro-inflammatory cytokines (such as TNF-α, IL-1β, etc.), forming a positive feedback loop [63]. It has been reported that NF-κB is highly expressed in skin lesions of psoriasis patients and highly activated in psoriatic keratinocytes [64]. The application of NF-κB antagonists can significantly reduce epidermal thickness, acanthosis, and inflammatory symptoms in psoriasis-like mice [8].Activation of the NF-κB signaling pathway induces the expression of pro-inflammatory factors such as NO, IL-1β, IL-6, and COX-2 [65]. Although it has been reported that the pathogenesis of psoriasis is related to TLR4/NF-κB or PI3K/AKT signaling pathway [65–68], the mechanism of action of PLE in the treatment of psoriasis has not yet been reported. Our previous studies have reported that leaf extract has a therapeutic effect on psoriasis [14], but its mechanism of action is unknown. Therefore, in this study, we found for the first time that the extract of the leaf can inhibit both TLR4/NF-κB and PI3K/AKT signaling pathways.

Studies have shown that PI3K/AKT is a key signaling pathway for cell survival and proliferation, and activation of this pathway promotes psoriasis onset and progression [69]. Various immune-mediated inflammatory and hyperproliferative skin diseases are also associated with dysregulation of the PI3K/AKT signaling pathway, and its activation also promotes the secretion of inflammatory cytokines [70,71]. Research shows that compared with normal skin, PI3K activity in epidermal lesions of patients with psoriasis increases 6.7 times. In addition, phosphorylated AKT is also strongly expressed in psoriatic skin lesions [72]. Activation of PI3K/AKT promotes the occurrence and progression of psoriasis, while inhibiting them attenuates the excessive proliferation of KC and the expression of inflammatory factors in psoriasis [73]. In addition, TNF-α also causes ROS generation and activation of PI3K/AKT, which subsequently triggers the activation of NF-κB, thereby regulating gene expression and inducing innate and adaptive immune responses and inflammation [74]. Activation of the PI3K/AKT signaling pathway by IMQ results in elevated expression levels of the second messenger PIP3. PIP3, upon binding to the PH structural domain signaling protein AKT, facilitates its phosphorylation. Subsequently, the activated AKT initiates the activation of IκB kinase-α (IKK-α) [75]. Regarding the pathogenesis of psoriasis, some scholars believe that it is related to the TLR4/NF-κB signaling pathway [65,66], while some scholars believe that the pathogenesis of psoriasis is related to the PI3K/Akt signaling pathway [67,68]. Although our previous preliminary studies have shown that the extract of the leaf has an improving effect on IMQ-induced psoriatic dermatitis [14], it has been found that the PLE can reduce the levels of TNF-alpha and IL-6 inflammatory factors in the serum of psoriatic mice, but the specific mechanism is not clear. Therefore, in this study, we conducted in-depth research on PI3K/Akt and PI3K/Akt signaling pathways to clarify the molecular mechanism, which is also one of the innovations of this study.

In recent years, with the continuous development of network pharmacology technology and its application in Chinese medicine research, network pharmacology technology has been widely used in the process of disease and target research [76,77]. Although this study preliminarily produced the mechanism of action of leaf extract in the treatment of psoriasis, the active ingredients of leaf extract in the treatment of psoriasis and further molecular mechanisms have not been studied. Therefore, in the future, we will use network pharmacology technology to study the active ingredients of leaf extract in the treatment of psoriasis and further molecular mechanisms.

Our study acknowledges several limitations that warrant consideration. First, regarding model specificity, the IMQ-induced mouse model effectively mimics psoriasis-like inflammation but may not fully capture the heterogeneity of human psoriasis. Thus, future clinical studies are essential to validate these findings in human contexts. Second, concerning mechanistic scope, our focus on the TLR4/NF-κB and PI3K/AKT pathways might overlook contributions from other signaling pathways; therefore, multi-omics approaches will be incorporated in subsequent investigations to provide a more comprehensive understanding. Third, potential biases need addressing: measurement bias could arise as blinded assessments were not explicitly detailed in all analyses, although we confirm that histopathological and ELISA evaluations were conducted under blinded conditions. Selection bias is another concern, as BALB/c mice were selected for their sensitivity to IMQ, yet strain-specific responses might restrict the generalizability of the results. Lastly, while diet and environmental factors were controlled, genetic variability remains an unaddressed confounding factor. In summary, we recognize these limitations and plan to emphasize clinical validation, broaden mechanistic exploration, and implement stricter blinding protocols in future research.

## Conclusions

Psoriasis is an intractable skin disease which has seriously affected the quality life of patients. Herein, we have found the anti-psoriasis effect of the *B. papyrifera* leaves extract (PLE). In in vivo tests, PLE effectively alleviated IMQ-induced psoriasis-like lesions, reduced the area and severity index of psoriasis lesions, reduced epidermal hyperplasia, improved the changes in the levels of SOD and MDA induced by oxidative stress, and reduced the changes in the inflammatory cytokines TNF-α and IL-17A. In addition, PLE suppressed the TLR4/NF-κB and PI3K/AKT signaling pathways. Our findings suggest that PLE is effective in improving psoriasis-like symptoms, which may lay the foundation for the development of plant-derived drug for the treatment of psoriasis. However, we recognize that several challenges such as formulation, stability, and pharmacokinetics remain to be addressed before PLE can be considered a viable drug candidate.

## Supporting information

S1 File Raw images. (PDF)

S1 Data Raw data of Figures. (ZIP)
