# Supplementary figures and images for: Broussonetia papyrifera ameliorates imiquimod-induced psoriasis-like skin inflammation in mice by modulating the TLR4/NF-κB and PI3K/AKT signaling pathways
Source: PLoS One. 2025 May 7;20(5):e0322710. doi: 10.1371/journal.pone.0322710 (PMC12057870; doi:10.1371/journal.pone.0322710)

Fig.6A

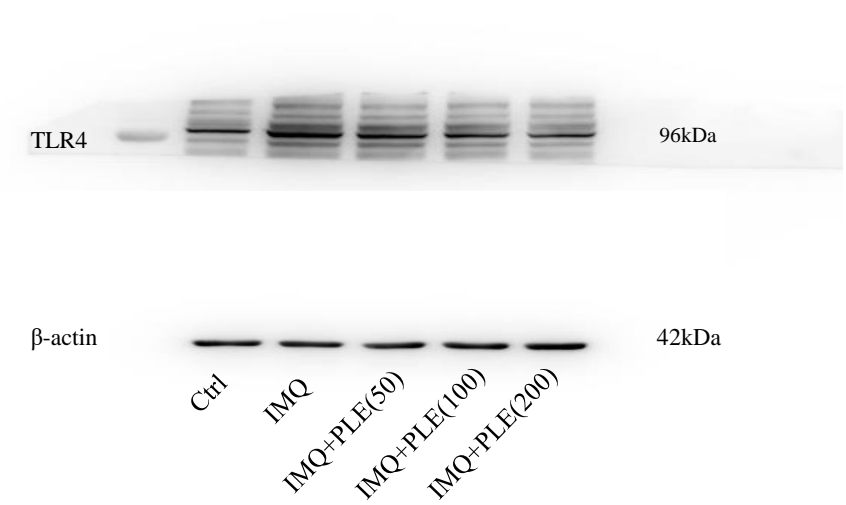

Fig.6B

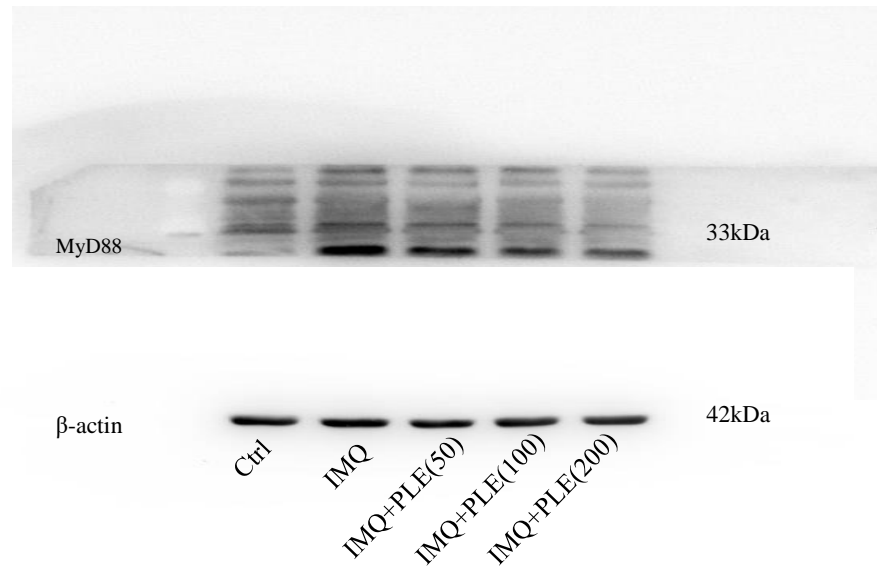

Fig.6C

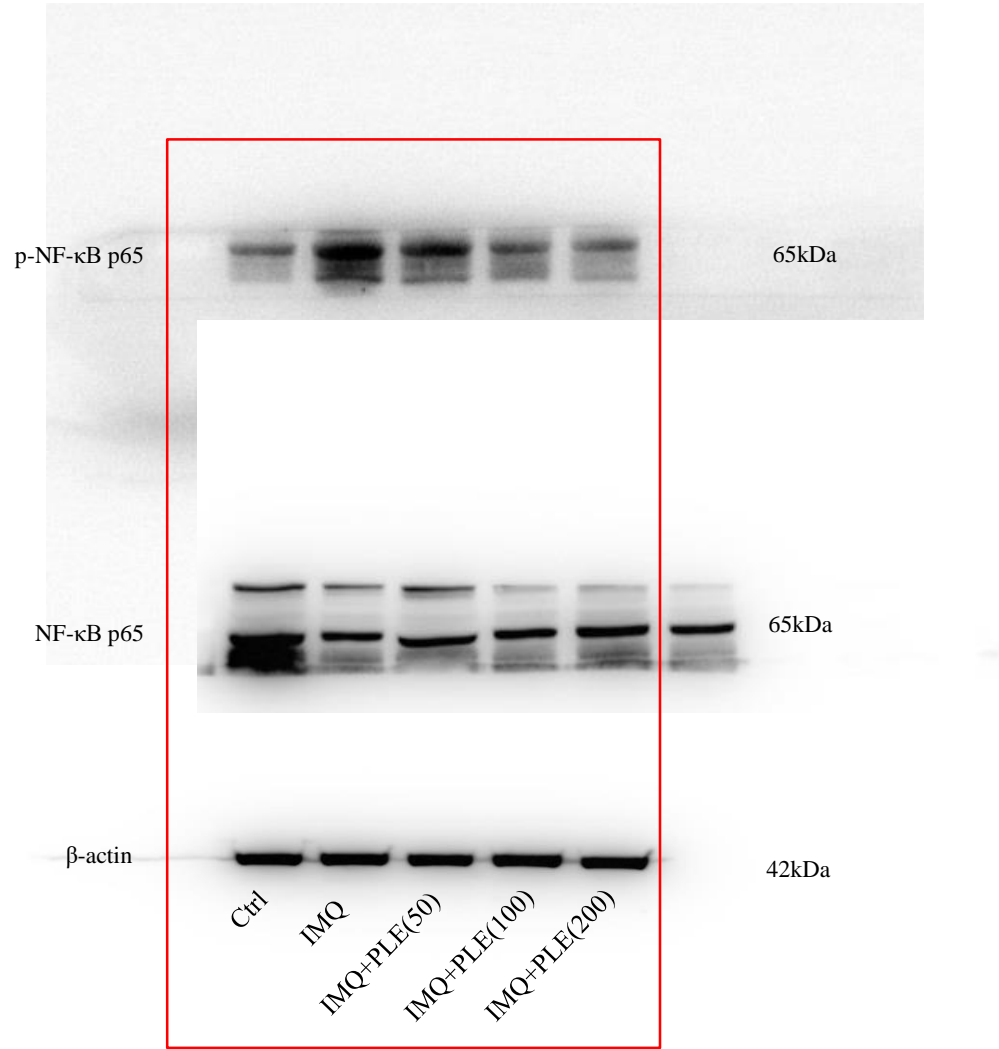

Fig.6D

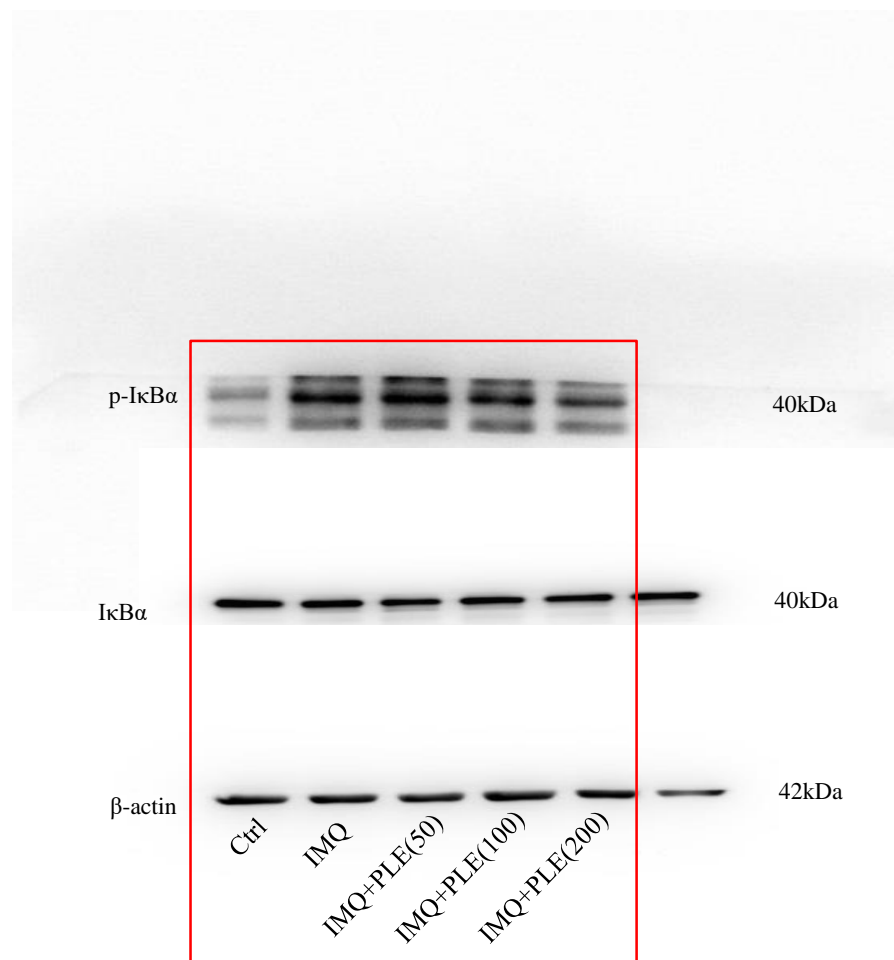

Fig.6E

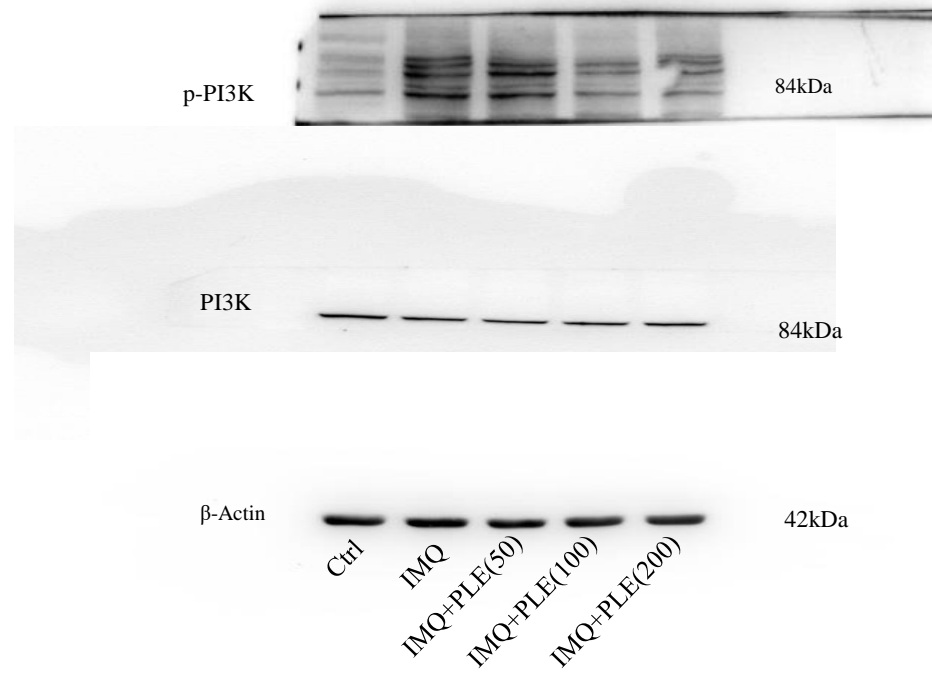

Fig.6F

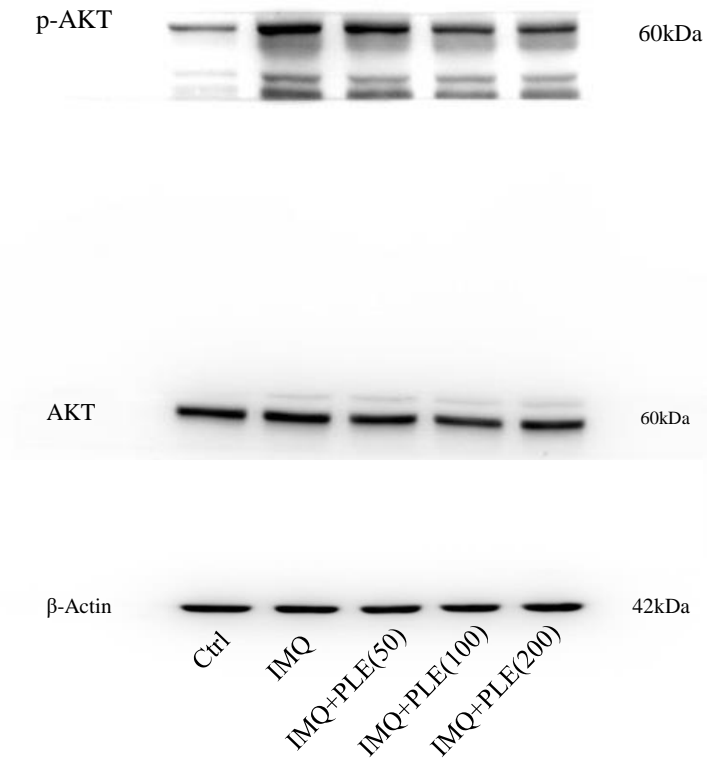

Supplement: S1 File — (PDF) [file pone.0322710.s001.pdf]
